# Unsupervised Machine Learning in the Evaluation of Telerehabilitation Interventions for Reading Fluency

**DOI:** 10.63144/ijt.2026.6735

**Published:** 2026-06-01

**Authors:** Chiara Pecini, Andrea Frascari, Viola Margheri, Kianna Kazemi, Pierluigi Zoccolotti, Gionata Manduchi

**Affiliations:** 1 Department of Education, Intercultures, Literature and Psychology (FORLILPSI), University of Florence, Florence, Italy; 2 Anastasis Social Cooperative Society, Bologna, Italy; 3 IFAB International Foundation Big Data and Artificial Intelligence for Human Development, Bologna, Italy; 4 Department of Psychology, Sapienza University of Rome, Rome, Italy; 5 Tuscany Rehabilitation Clinic, Montevarchi (Arezzo), Italy; 6 Gianfranco Salvini Foundation, Montevarchi (Arezzo), Italy

**Keywords:** Learning disorder, Machine learning, Reading, Telerehabilitation, Unsupervised learning

## Abstract

The implementation of machine learning techniques enables the analysis of large data corpora to differentiate response patterns based on exercise parameters, providing insights for implementing efficient telerehabilitation of reading skills. In this study, we applied unsupervised machine learning methods to investigate rehabilitation training trajectories in response to a self-adaptive teleintervention of reading decoding. We analyzed data from 6,692 children and adolescents using the Reading Trainer app for at least two months. Using K-means clustering, we identified eight distinct learning curve patterns, subsequently categorized as No-responders, Partial Responders, and High Responders based on the differences between initial and final reading performance. Multinomial regression analysis showed that younger children with greater initial difficulties and those who completed a higher number of in-session exercises during treatment were more likely to be classified as High Responders. These findings provide crucial insights to predict responses to reading intervention and help in personalizing telerehabilitation strategies.

In recent years, telerehabilitation has emerged as a promising approach to deliver rehabilitation services (Hill et al., 2025), including interventions on school learning skills (Ogourtsova et al., 2023), in part as a response to the school complexities associated with the COVID-19 pandemic (Cadime et al., 2024; Cruz et al., 2022; Verwimp et al., 2024). It provides new opportunities to overcome logistical challenges, reduce waiting times, and enable early intervention (Hill et al., 2025). Beyond these organizational benefits, digital environments facilitate the administration of exercises that are both intensive and personalized. These factors could be crucial in promoting the automation of learning processes for children with reading disorders (Casalini, 2024).

## Reading Difficulties

Challenges in acquiring essential learning skills, such as reading, spelling, and mathematics, can be categorized as either specific or nonspecific. Specific Learning Disorders (SLD) refer to persistent difficulties in mastering and automating basic academic skills while excluding general cognitive deficits or adverse environmental factors (DSM-5, 2013). On the other hand, nonspecific learning difficulties originate from socio-cultural disadvantages or atypical developmental conditions, such as language disorders, attention-deficit/hyperactivity disorder (ADHD), autism spectrum disorders (ASD), or more complex neurological issues like neuromuscular disorders or cerebral palsy. Overall, both specific and nonspecific learning difficulties impact over 20% of the school population and are a significant risk factor for school dropout (Schleicher, 2019), with lasting effects on quality of life in adulthood.

Reading disorder is one of the most common issues, involving difficulties in accurately and fluently decoding and/or comprehending written text. According to the most acknowledged framework, reading requires several processes, including decoding and language comprehension (Gough & Tunmer, 1986). It also involves ‘bridge processes,’ like phonological and morphological awareness and executive functions, which support the connection between word recognition and meaning (Duke & Cartwright, 2021). Some processes, such as comprehension strategies, become progressively more strategic during development. In contrast, others, including word decoding (Scarborough, 2001), require high automatization. To automate decoding, attentional, visual, and linguistic processes must mature and coordinate from an early age. This development depends on sublexical strategies for converting graphemes into phonemes, which later integrate with lexical access strategies (whole word recognition), essential for fluent reading (e.g., Orsolini et al., 2006; Share, 1995). Due to the complexity of acquiring decoding skills, reading disorders can have multiple causes and present in various forms, necessitating early and highly individualized intervention strategies. By facilitating the automatization of decoding, these interventions can free cognitive resources for higher-level comprehension.

The scientific literature demonstrates substantial variability in the outcomes of interventions for reading difficulties, which are influenced by the underlying type of deficit, the age of initial support, and the specificity of the training approach (e.g., phonological instruction compared to lexically based strategies). Interventions that directly address alphabetic–phonological and orthographic processes typically produce the most robust improvements in word decoding and spelling, while less targeted methods or broad cognitive programs yield weaker effects (Galuschka et al., 2020; Toffalini et al., 2021). The effectiveness of these programs varies substantially, even among established approaches such as phonics, highlighting the importance of tailoring intensity, instructional focus (i.e., phonology, orthography, or fluency), and sensory support to match individual learner profiles (Hall, 2023). Recent studies employing neurophysiological measures and eye-tracking indicate that visual adaptations or targeted exercises focused on attentional dynamics and orthographic processing can further enhance outcomes and facilitate the transfer of learning (e.g., Toki et al., 2024). To optimize intervention effectiveness and mitigate long-term academic and psychological consequences, it is necessary to develop flexible, personalized intervention models that are adapted in terms of intensity, targets, and delivery formats. Delayed or ineffective training in decoding skills may lead to additional difficulties with other essential skills and adversely impact educational and vocational opportunities, thereby increasing the risk of comorbid psychological disorders over time.

## Telerehabilitation of Reading Disorders

Incorporating outpatient exercises with independent activities carried out at home through telerehabilitation allows for timely, daily interventions for reading difficulties, while maintaining the quality of the exercises. These can be continuously monitored and adjusted based on the child’s responses, reducing the risks associated with delayed or ineffective training (Del Lucchese et al., 2024).

Over the past 10 to 20 years, various telerehabilitation platforms and apps have focused on reading decoding. Some aimed to enhance the underlying cognitive processes (e.g., Cancer et al., 2021; Capodieci et al., 2022; Pecini et al., 2019), while others concentrated on the decoding task itself (e.g., Kohnen et al., 2021; Pecini et al., 2018; Tressoldi et al., 2012; Tucci et al., 2015; Verwimp et al., 2024). The cognitive process-focused apps engage users in activities that aim to improve visual attention, visual-verbal integration, and rhythm perception, essential factors for fluent reading. Conversely, decoding-oriented applications require users to practice reading aloud syllables, words, or texts of varying difficulty. These exercises progressively increase speed and include phonological and lexical reinforcement instruction based on established research findings (Torgesen et al., 2006; Zoccolotti et al., 2009). Notably, some programs combine both types of training (e.g., Capodieci et al., 2023; Lorusso et al., 2022; 2024a; 2024b). In terms of feasibility and usability, several studies have emphasized how telerehabilitation decoding trainings are associated with positive impact and satisfaction expressed by the child and parents (Lorusso et al., 2024b), cost reduction and increased training efficiency (Tressoldi et al., 2012), training sustainability (Capodieci et al., 2022; Lorusso et al., 2024a), and waiting list reduction (Verwimp et al., 2024).

The increasing popularity of teleintervention programs has fostered the development of numerous experimental investigations. These studies consistently show that intensive, self-directed training conducted at home under remote supervision significantly improves both the speed and accuracy of reading words, non-words, and texts (Capodieci et al., 2023; Lorusso et al., 2022, 2024a, 2024b; Kohnen et al., 2021; Pecini et al., 2018, 2019; Tressoldi et al., 2012; Tucci et al., 2015). Additionally, these programs have a positive impact on other reading and writing skills (Cancer et al., 2021; Eroğlu et al., 2022), with no significant differences compared to in-person trainings (Cancer et al., 2021; Lorusso et al., 2024b; Verwimp et al., 2024). Among the telerehabilitation programs for reading decoding that align with the aforementioned characteristics, a widely utilized intervention in the Italian context is Reading Trainer (Anastasis Coop, 2013). Through timed text-reading exercises, this program aims to improve oral reading fluency. The intervention has proven effective on decoding fluency across various samples of children diagnosed with developmental dyslexia (Tucci et al., 2015), regardless of the severity of their literacy difficulties (Pecini et al., 2018). In addition, its efficacy has been demonstrated in comparison to passive control groups and in combination with interventions targeting the underlying cognitive processes of reading (Capodieci et al., 2023).

While the results are promising, the studies vary significantly in their populations, which differ in age and severity of the disorder. Additionally, the types of training administered and the methodologies employed are different from one study to another. For instance, it remains unclear how the intensity and duration of interventions, along with the integration of various exercise techniques, may impact their effectiveness. Furthermore, most studies rely on pre-post designs that do not effectively account for the intervention’s performance and are subject to variable measurement errors (Evans et al., 2017).

Telerehabilitation methodologies offer a significant opportunity to overcome the limitations of traditional pre-and post-analyses by enabling continuous and automated monitoring of a child’s performance. By collecting longitudinal and multidimensional data, these methods allow for real-time visualization of intervention progress, supporting clinicians in evaluating the effects of their intervention and determining the most effective timing for adjustments. Additionally, this approach helps identify individual response patterns through clustering and machine learning techniques, which categorize profiles based on behavioral sequences that can predict performance (Gašević, 2017).

Machine learning is a field of artificial intelligence focused on developing algorithms capable of learning from data without being explicitly programmed to perform a specific task (Sinaga & Yang, 2020). The primary goal of this discipline is to create computational models that can identify meaningful patterns within data, make predictions on new inputs, or support decision-making based on the information learned. Unsupervised learning operates on unlabeled data, aiming to uncover intrinsic patterns, structures, or groupings within the data itself. This type of learning is beneficial for initial data exploration and for revealing hidden dimensions or categories. Typical techniques include clustering algorithms (such as k-means and hierarchical clustering) and dimensionality reduction methods (such as Principal Component Analysis – PCA) (Bishop, 2006; Hastie et al., 2009; Jolliffe & Cadima, 2016). Moreover, when integrated with individual and environmental characteristics, this analysis lays the groundwork for personalized education and more tailored training (Tilanus et al., 2019). In clinical settings, this is relevant for optimizing training duration, avoiding premature discontinuation for those who experience a rapid plateau, and encouraging continued training for individuals who show slow but consistent improvement. Clustering of the response to training has been recently applied to telerehabilitation (Antoniello et al., 2023). Nevertheless, to date, no studies have been conducted on the telerehabilitation of children with reading difficulties.

To align with the principles of personalized rehabilitation medicine and create clearer operational guidelines for addressing reading difficulties as a function of the developmental stage, it is relevant to examine how individual responses to telerehabilitation training vary in children and adolescents with decoding difficulties. Additionally, it is also important to investigate whether different learning profiles are associated with specific individual characteristics and training methods.

## Objectives and Hypotheses of the Study

In this study, we examined the learning progression of Italian children with reading fluency disorders who used the Reading Trainer (https://www.anastasis.it/ridinet/app-ridinet/reading-trainer/, Tressoldi, 2013), an application within the RIDInet platform for integrated telerehabilitation of learning disorders (Anastasis Coop, 2013; Capodieci, 2024). This study adopts a retrospective design, with data collected after the completion of the training program.

Italian is a language with a highly regular orthography in the grapheme-to-phoneme direction, while moderately irregular in the phoneme-to-grapheme direction (Burani et al., 2017). In regular orthographies, reading difficulties are expressed primarily in terms of reading slowness, while accuracy may be near-normal or only moderately impaired (Wimmer, 1993; Zoccolotti et al., 1999).

Reading Trainer aims to treat reading difficulties through clinician-adjusted, auto-adaptive parameters that aim to improve fluency in decoding, taking into consideration the individual’s level of accuracy (Tressoldi, 2012). Group studies have examined the effectiveness of Reading Trainer in increasing the reading speed of children (Pecini et al., 2019; Tucci et al., 2015) and adolescents (Pinnelli, 2014) with dyslexia.

The present study applied unsupervised learning procedures to data collected with Reading Trainer with three aims:

- A1. Clustering the curve of learning in response to a telerehabilitative intervention for reading decoding in children and adolescents with reading difficulties.
- A2. Verifying whether individual variables, such as age, initial reading level, and number of exercises, differentiate the learning curves.
- A3. Verifying whether some crucial parameters of teleintervention, i.e., types and modes of exercise, differentiate the learning curves.

The following hypotheses were formulated:

- H1. The learning responses to tele-training of decoding difficulties may cluster according to different time trajectories, which reflect differences in the gains obtained from the beginning to the end of the training.
- H2. Different groups of learning curves vary by the child’s age, difficulty level, and number of exercises. According to the literature on reading intervention, we expected that younger children with lower performance, and/or following an intensive intervention, would have higher chances of modifiability than older children with mild impairments and/or following non-intensive training.
- H3. Different groups of learning curves vary according to the training parameters, with higher gains when exercises are tuned to individual performances during training.

The study was approved by the Ethics Committee of the University of Florence (approval no. 282, October 20, 2023).

## Method

### Training Characteristics

**Figure 1:**
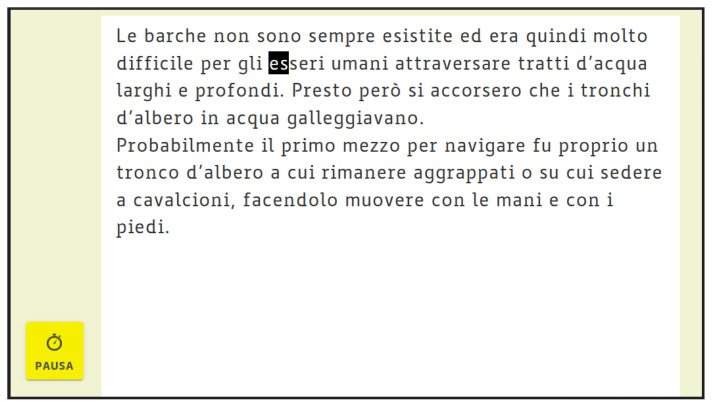
Example of a Text Used During the Reading Trainer Exercise (Syllable Reading Unit)

Data were collected using the Reading Trainer (Tressoldi, 2013), an application within the RIDInet platform for integrated telerehabilitation of learning disorders (Anastasis Coop, 2013). Reading Trainer is an evidence-based program envisaging clinician-adjusted, auto-adaptive parameters for improving reading fluency, in terms of both accuracy and rate (Tressoldi, 2012; Tucci et al., 2015; Pecini et al., 2018, 2019; Capodieci et al., 2023). Based on the Dual-Route Model of reading (Coltheart et al., 2001), it aims to shift the reading process by progressively decreasing the presentation time of stimuli (at both the word and syllable levels). This time-pressure mechanism aims to force direct lexical access to the word, thereby discouraging the reliance on slower grapheme-to-phoneme conversion processes (the non-lexical route). The child must read aloud a digital book selected from an extensive library, with the supervision of an adult. During the activity, reading units (i.e., graphemes, syllables, morphemes, or words) are highlighted in a karaoke-like format (see Figure 1) and paced according to the child’s reading speed. The child is required to maintain the pacing, while the supervising adult records reading accuracy errors at the end of each text. These errors include typical deviations in reading accuracy, such as omissions, insertions, substitutions, and other comparable error types. As reading accuracy errors are not automatically coded by the platform, their documentation is the responsibility of the supervising adult. The supervising adult may be either a trained clinician (e.g., a speech and language therapist or psychologist with specific training in the use of the Ridinet telerehabilitation platform) or a parent/caregiver, depending on whether the session is conducted in a clinical setting or in a home-based context.

The study baseline was defined by the results of the first 10 exercises. Specifically, for each participant, the baseline comprised three variables. *Initial accuracy* and *initial speed* were calculated as the mean of the data recorded during the first 10 exercises. *Reading difficulty level* was defined as the standardized value of initial speed relative to the participant’s grade level, based on normative data from the MT reading tests (Cornoldi et al., 2017).

The standard intervention typically lasts between two and three months and consists of 20-minute sessions conducted four times per week. These sessions take place both in the clinician’s office and at home, with the involvement of family members who support the intervention by facilitating technical access to the platform, monitoring the child’s participation and ensuring the correct completion of the activities, as well as providing motivational support when needed. The clinicians train parents in the use of the software and in the procedures required to support the training. Parents are involved in the administration and supervision of the training procedures, but do not provide direct instructional prompts during task performance. Before the intervention, clinicians establish individualized treatment goals based on each child’s functional reading profile, as assessed by a clinician through standardized measures of reading speed (measured in syllables per second) and accuracy (calculated as the percentage of correctly read units out of the total number of units in the text). This procedure is consistent with previous Reading Trainer protocols (e.g., Tucci et al., 2015; Pecini, 2019). For the present study, although clinical assessments were conducted routinely before and after intervention, the corresponding standardized assessment data were not available. This was because participants were followed by multiple clinicians across various clinical settings. Consequently, for the present analyses, baseline and outcome levels were determined using the initial and final performance measures recorded within Reading Trainer.

The targets are pursued through an adaptive learning mechanism. Once a child successfully reads a predetermined number of texts, defined as achieving an accuracy rate above the established threshold, the reading speed is automatically increased by 0.1 syllables per second.

The intervention permits multiple configuration options to accommodate each child’s reading profile. Personalization is achieved through adaptive or manual adjustments of the following parameters:

- Reading unit: The child reads pre-defined textual units (scanned), which may be set at sublexical (i.e., syllables, morphemes) or lexical level (i.e., words).
- Progression mode: In timed mode, reading speed (i.e., text scanning rate) increases automatically when the child meets the clinician-defined accuracy threshold across at least three consecutive texts. In manual mode, the child initiates progression by pressing the space bar or tapping the screen.
- Page length: The clinician selects texts according to the child’s age and attentional capacity, with lengths ranging from very short to very long.
- Presentation mode: Texts can appear in one of four ways: (a) full text, (b) previous text (the text read and the current unit), (c) remaining text (the current unit and subsequent text), or (d) individual units (only the current unit).

The presentation rate of the reading unit increases automatically by 0.1 syllables per second after a series of exercises completed at the target accuracy level. In contrast, other parameters, such as page length, reading unit, and advancement mode (manual or timed), are set by the clinician based on the child’s reading profile. In the present study, we did not include the number of parameter changes among the independent variables; however, it is worth noting that the data showed substantial variability across participants (M = 11.7, SD = 13.2).

Based on the analysis of each child’s reading profile before the intervention (e.g., Luci et al., 2022; Tucci et al., 2015), the clinician selects the most appropriate parameter configuration. For every user, session, and exercise, both the applied parameters and the performance metrics are automatically recorded. The recorded parameters include the number of exercises per session, the reading unit applied, the type of progression mode, and the text length. The performance measures included reading speed, expressed in syllables per second, and reading accuracy, calculated as the percentage of correctly read units (e.g., syllables, words, morphemes) out of the total number of units in the text.

Training sessions conducted for at least 2 months, and an average of 4 sessions per week, were selected. The intervention consisted of 20-minute sessions (including breaks) conducted approximately four times per week over a period of two to three months. However, as they represent general guidelines provided by the App, clinicians are free to adjust the duration based on the clinical profile of each child, with the session duration correlated to the number of exercises. Thus, the intensity of the training is not fixed, and the actual number of exercises completed may vary considerably.

The collected data indicate that the average effective duration was 13 net minutes. It is also worth noting that the intervention consistently began and ended in the clinician’s office. However, the number of additional sessions conducted in the clinician’s office was not predetermined, resulting in a significant portion of the telerehabilitation being carried out at home under the supervision of a caregiver.

### Sample

For the present study, data were collected from 2017 to 2023 on 6,692 children aged between 7 and 14 years (mean age = 8.60, SD = 1.36) who attended public and private centers for reading disorders. No additional developmental, psychiatric, or sensory disorders were available in their clinical records.

Parents gave consent to the processing of data in an anonymous form for scientific research purposes. For the sake of the study’s aims, we adopted no exclusion criteria related to the diagnostic condition or functional profile of the participants.

### Data Analysis

#### Learning Curve Clustering (LC-clusters)

Raw reading speed (syllables/second) from each training session was organized into daily time series for each participant: *t*_1_, *t*_2_, … , *t_n_* where n = 60 days corresponding to the intervention period. Each daily value represents the average reading speed across all exercises completed on that day, enabling the analysis of individual trajectories.

The dataset was cleaned to remove sessions affected by systematic or random errors. Individual exercise sessions were excluded based on the following criteria:

- Spikes in reading speed exceeding 50% of the previous session’s value, inconsistent with the participant’s prior performance trajectory,
- Duplicate readings of the same page within a single exercise,
- Reading speeds exceeding five syllables/second, are deemed incompatible with the typical developmental ranges for the child’s age group.

Missing day values were imputed using linear interpolation based on the nearest preceding. To reduce short-term fluctuations and noise in the time series, a three-point moving average filter was applied to smooth the learning trajectories while preserving the underlying trend.

**Figure 2:**
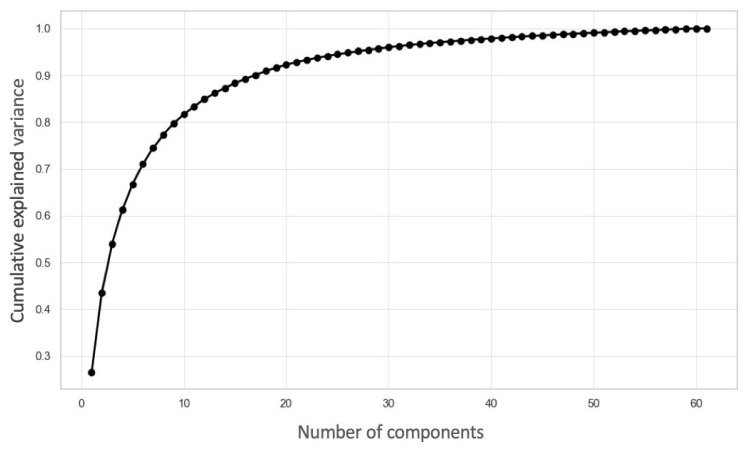
Cumulative Explained Variance by Number of Components

We implemented unsupervised machine learning procedures using Python (Python 3.12 with libraries NumPy 1.26.4, Pandas 1.3.5, and Sklearn-pandas 1.8.0). To manage the high-dimensional nature of the 60-day time series data, Principal Component Analysis (PCA) was applied for dimensional reduction. PCA transforms the original correlated variables into a smaller set of uncorrelated components that capture the essential patterns of variation in learning trajectories. The number of components retained was determined by examining the cumulative explained variance. Sixteen components were selected, collectively accounting for approximately 90% of the total variance in the original dataset (see Figure 2), balancing information preservation with computational efficiency for subsequent clustering analysis.

To identify distinct patterns in learning trajectories, we applied K-means clustering to the 16 principal components derived from PCA. The K-means algorithm partitions observations into k clusters by iteratively minimizing the within-cluster sum of squared distances while maximizing between-cluster separation. This unsupervised approach enabled the identification of user subgroups exhibiting similar development trajectories without prior knowledge of group membership. The optimal number of clusters was determined using the elbow method. Results indicated that k = 8 clusters provided the best balance between model complexity and explained variance. To verify cluster stability, we computed silhouette coefficients across solutions ranging from k = 4 to k = 12. The k = 8 solution yielded the highest mean silhouette score and the most consistent cluster assignments across resampling iterations, supporting its selection.

### Parameters Clustering

Beyond trajectory-based clustering, we examined two additional parameters to characterize learning patterns: (1) *reading unit* (RU-Clusters) and (2) *progression mode* (PM-Clusters). To explore whether these parameters exhibited distinct patterns across participants, we performed separate K-means clustering analyses on the RU and PM data following the same procedure described above. This multi-level clustering approach allowed us to examine both temporal learning dynamics (trajectory clusters) and qualitative learning characteristics (RU and PM clusters).

#### Cluster Differences

We conducted multivariate inferential statistical analyses using the open-source software Jamovi (Version 2.6.44.0) and Python scripts (*Python 3.12, NumPy 1.26.4, Pandas 1.3.5*, and *Sklearn-pandas 1.8.0*). The normality of the distribution of the dependent variables was assessed based on skewness and kurtosis values (both < 2). We tested the homogeneity of variances using the Levene test (p > .05). To determine each participant’s reading speed and accuracy at the beginning and end of the training, we selected the first 10 and last 10 exercises (Beginning and End training, respectively, B–E); then, we computed the means of syllables per second and the ratio of correctly read units to the total number of reading units. To standardize the difficulty level at the beginning of the intervention, the initial reading speed was transformed into z-scores based on national age-normed means (initial reading difficulty).

To confirm the significance of the machine learning results, we conducted mixed-design ANOVAs on the mean reading speed and accuracy at the beginning (B) and end (E) of the training as a within-subjects variable and the Learning curve (LC) cluster membership as a between-subjects variable. Based on the results obtained (see below), the LC-clusters were re-classified into three groups: No-responders, Partial responders, and High-responders. To investigate how the child’s characteristics and behavior (i.e., age, initial reading speed, initial reading difficulty level, number of exercises) contributed to explaining membership to different Cluster groups, a multimodal logistic regression analysis was used (Hosmer et al., 2013). We treated age as a continuous variable in all analyses. To investigate how RU and PM clusters interacted with the training gains, two mixed-design ANOVAs were run on reading speed with B–E training as a within-subjects factor and PM-Cluster and RU-Cluster as between-subjects factors, respectively. Given the large sample size, we interpreted the results of the inferential analyses based on effect size. Standard cutoffs were used for partial η^2^ (from 0.01 for a small effect, 0.06 for a medium effect, and 0.14 for a large effect) and Cohen’s d (from 0.2 for a small effect, 0.5 for a medium one, and 0.8 for a large one) (Cohen et al., 2013).

## Results

The final dataset used for the cluster analysis comprised 922,467 individual training exercises completed by 6,692 children undergoing the Reading Trainer training. We extracted the number of exercises from the training logs and treated it as an independent variable. Although the instructions given by the clinicians recommended completing approximately one session every two days, the total number of exercises depended on the session duration (a parameter determined by the clinicians) and on the child’s actual adherence to the prescription. Raw reading speed showed high inter-subject variability across the 60 time points, such as absence of changes, initial increases followed by plateaus, or initial plateaus followed by final increases.

### Learning Curves

**Figure 3:**
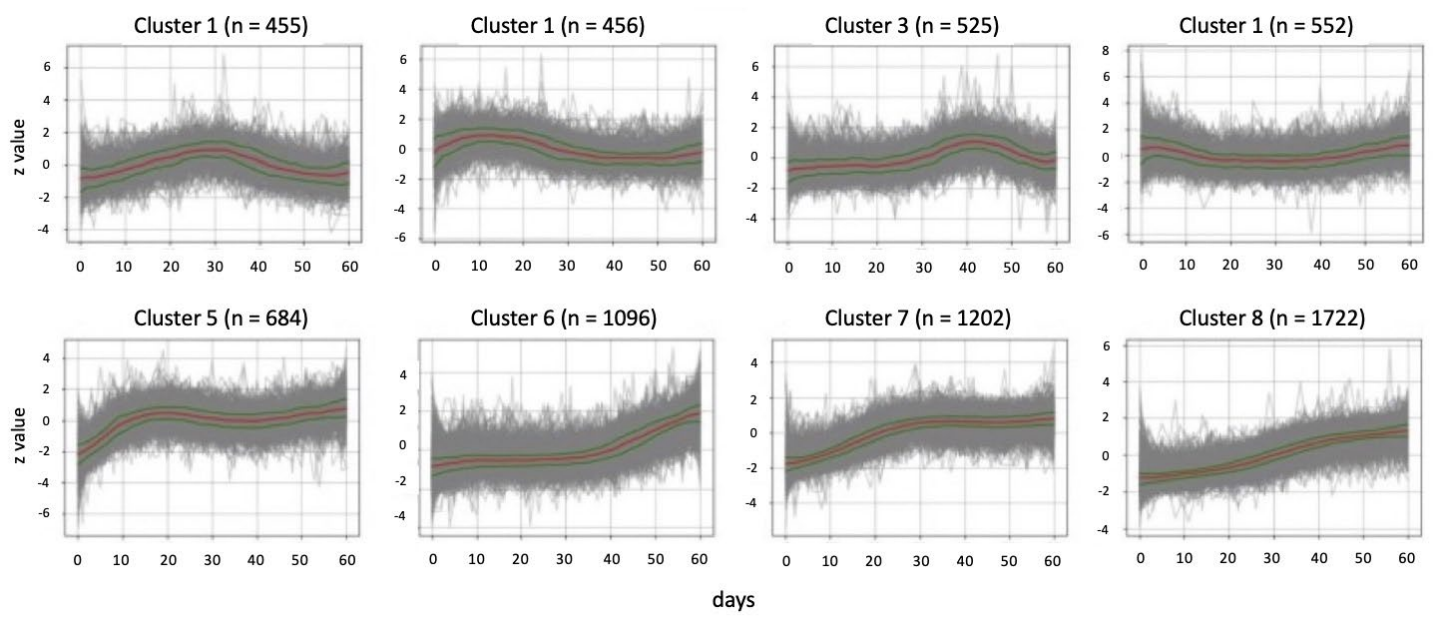
LC Clusters of Reading Speed Learning Curves over the Course of Training (Cluster Sizes are Reported in Each Panel) *Note*. The red line represents the mean learning trajectory for each cluster, while the green lines represent the 25th and 75th percentiles across participants within each cluster.

K-means clustering based on *learning curves* in reading speed during the training time resulted in 8 distinct learning curves (LC-Cluster, Figure 3). Each cluster represents a group of individuals sharing common characteristics in terms of progress in reading speed. Visual inspection showed that LC-Clusters 1–4 (n = 455, 456, 525, 552, corresponding to 6.80%, 6.81%, 7.85% and 8.25% of the total sample, respectively) had a variable learning trend without a clear tendency; LC-Cluster 5 (n = 684; 10.22%) showed an increase constrained to about the first two weeks of training; and LC-Clusters 6–8 (n = 1096, 1202, 1722; corresponding to 16.38%, 17.96% and 25.73% of the total sample, respectively) were characterized by an appreciable increase over the entire intervention period, although with different slopes. Specifically, LC-Cluster 6 experienced a greater enhancement during the latter half of the training; LC-Cluster 7 saw more improvement in the initial phase; and LC-Cluster 8 maintained a consistent increase throughout the entire training duration.

### Parameter Clustering

**Figure 4:**
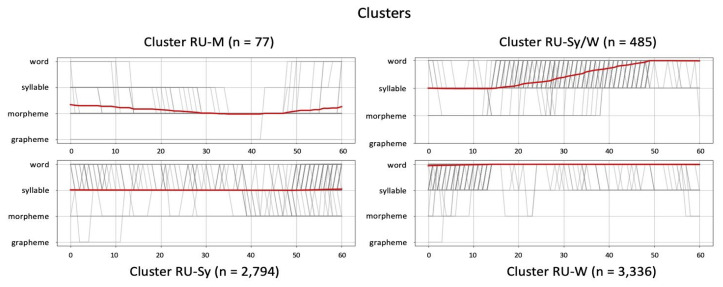
Clustering Based on the Reading Unit Parameter (RU), Expressed in Graphemes (Cluster Sizes are Reported in Each Panel) *Note*. The y-axis represents reading unit (RU) categories (1 = morphemes; 2 = syllables/words; 3 = syllables; 4 = words). The red line indicates the median number of children who had the training configured in a given mode on a specific day.

K-means cluster analysis based on the *reading unit* parameter identified four distinct clusters (RU-Cluster, Figure 4). RU-Cluster 1 (n = 77): morphemes (RU-M); RU-Cluster 2 (n = 485): syllables switched to words (RU-Sy/W); RU-Cluster 3 (n = 2,794): syllables (RU-Sy); and RU-Cluster 4 (n = 3,336): words (RU-W).

**Figure 5:**
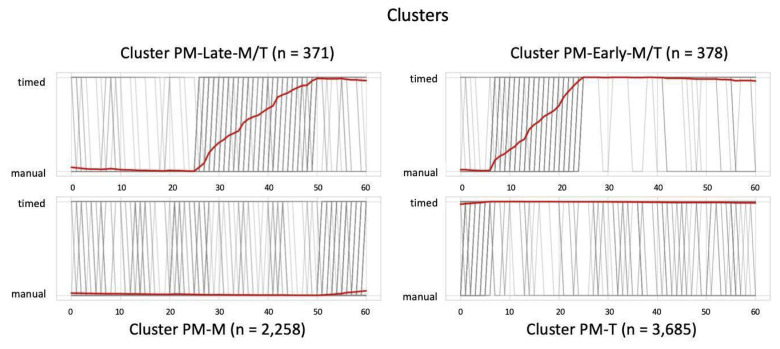
Clustering Based on the Progression Mode Parameter (PM-Clusters; Cluster Sizes are Reported in Each Panel) *Note*. The y-axis represents training mode (1 = manual mode; 2 = timed mode). The red line indicates the median proportion of children assigned to each training mode across time. For example, in Cluster PM-Late-M/T, all children were in manual mode up to day 25, and all were in timed mode from day 50 onward; between these time points, a gradual transition occurred (e.g., by day 40, approximately 70% had transitioned).

Clustering based on the *progression mode* (PM-Cluster, Figure 5) identified four distinct clusters. PM-Cluster 1 (n = 371): from manual and timed modes between the 25th and the 50th day of training (PM-Late-M/T); PM-Cluster 2 (n = 378): from manual to timed mode between the 6th and 23rd day (PM-Early-M/T); PM-Cluster 3 (n = 2,258): always in manual mode (PM-M); and PM-Cluster 4 (n = 3,685): always in timed mode (PM-T).

### LC Clusters Differences

**Figure 6:**
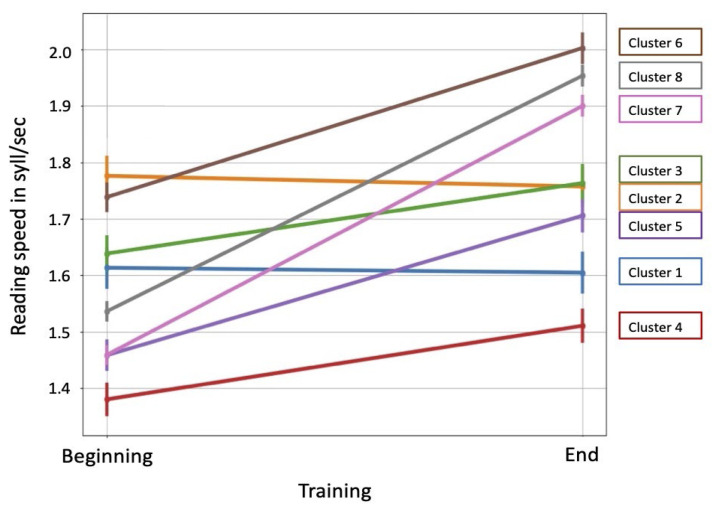
Means and Standard Errors of the Reading Speed Raw Scores Obtained at the Beginning and End of the Training in the 8 LC-Clusters

A mixed ANOVA was conducted on reading speed, with B-E (beginning vs. end of training) as the within-subjects factor and LC-cluster membership as the between-subjects factor. The analysis revealed a significant main effect of B–E training, F _(1,6575)_ = 3305.00, p < .001, partial η^2^ = .334, indicating a large effect size. In addition, the B–E training × LC-cluster interaction was significant, F _(7,6575)_ = 396.00, p < .001, partial η^2^ = .296, showing that the differences between initial and final reading speed varied significantly across LC-clusters (see Figure 6).

Post-hoc analyses (Tukey test) did not show significant differences between the beginning and end of the training for LC-Clusters 1 and 2. In contrast, differences were significant for LC-Clusters 3 to 8 (Table 1), although with variable effect sizes.

Regarding reading accuracy, the initial mean accuracy in all clusters ranged from 96.3% to 97.1%. A mixed-design ANOVA was conducted on accuracy, with B–E (beginning vs end of training) as the within-subjects factor and LC-cluster membership as the between-subjects factor. Despite statistical significance of the main effect of B–E training, F _(1,14786)_ = 166.0, p < .001, partial η^2^ = .025, and of the B–E training × LC-cluster interaction, F _(1,14786)_ = 12.9, p < .001, partial η^2^ = 0.014, the small effect size and the mean differences observed in the post hoc comparisons (maximum of 0.7% between clusters 1 and 8) suggest small changes across the whole intervention and absence of differences across the LC-Clusters on reading accuracy.

**Figure 7:**
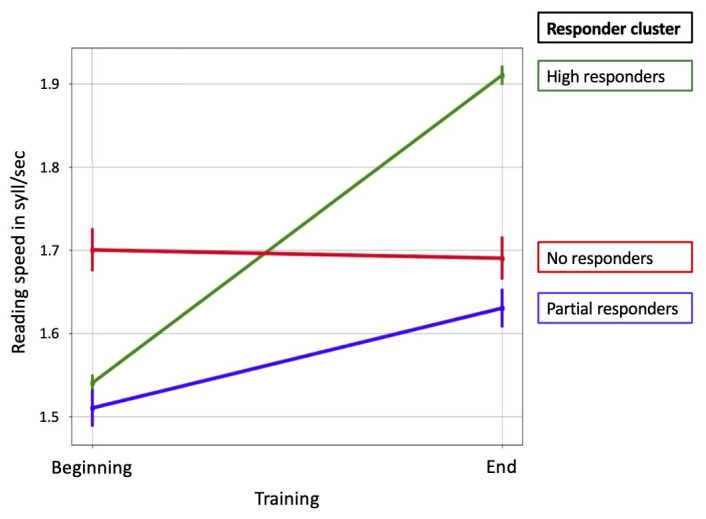
Means and Standard Errors of the Reading Speed Scores Obtained at the Beginning and End of the Training in the No, Partial, and High Responders Clusters

Considering reading speed, based on the mean differences between the beginning and end of the training and the values of the effect size (Table 1), the 8 LC-Clusters were collapsed into three groups of subjects: No-responders (NR, corresponding to LC-Clusters 1–2), Partial-responders (PR, corresponding to LC-Clusters 3–4), and High-responders (HR, corresponding to LC-Clusters 5 to 8). Descriptive statistics of the reading speed scores for each of the three groups are presented in Figure 7.

We conducted a multinomial logistic regression to examine how children’s characteristics and behavior (i.e., age, initial reading accuracy, initial reading speed, initial reading difficulty, number of exercises) predicted membership in the three groups (No, Partial, High responders). The model was split to evaluate its performance on a test set. Cross-validation was then performed to obtain a more robust estimate of its predictive ability. The overall accuracy of the model is 0.69, indicating that about 69% of the total predictions were correct, although with a high variability across groups.

- No responder: The model had a precision of 40% for this class, but a very low recall of 3%, suggesting it rarely predicted this class, and when it did, it was right 40% of the time. Precision: 0.40; Recall: 0.03; f1-score: 0.05.
- Partial responder: The model showed 0% precision and recall for this class, indicating it completely failed to predict any samples as ‘Partial responder.’ Precision: 0.00; Recall: 0.00; f1-score: 0.00.
- High responder: The model performed very well for this class with 70% precision and 100% recall, meaning it correctly identified all ‘High responder’ samples. Precision: 0.70; Recall: 1.00; f1-score: 0.82.

**Figure 8:**
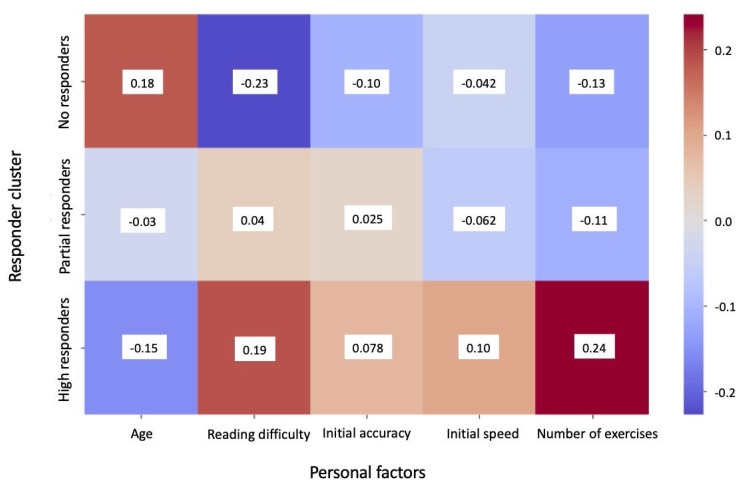
Heatmap of the Results of a Multinomial Logistic Regression Predicting Cluster Membership (No, Partial, and High Responders) from Children’s Characteristics (Age, Reading Difficulty, Initial Reading Accuracy, Initial Reading Speed, and Number of Exercises) *Note*. The y-axis represents cluster membership.

The results, graphically represented by a heatmap (James et al., 2021; Wilkinson & Friendly, 2009), show variability in the log-odds coefficients, representing the strength of the relationship between the predictors and group membership (Figure 8).

Age was a positive predictor of group membership, such that older children were more likely to belong to the No-responders group (β = 0.15). In contrast, younger children were more likely to belong to the High-responders group. Initial reading difficulty was negatively associated with the No-responder membership (β = −0.23) and positively with the High-responders membership (β = 0.19), indicating that beginning the intervention with greater difficulty increased the likelihood of belonging to the High-responders rather than the No-responders group. Initial reading accuracy had a negligible effect across all clusters. Finally, the number of exercises completed positively predicted membership in the High-responders group (β = 0.24) and negatively predicted membership in the Partial-responders (β = −0.11) and No-responders (β = −0.13) groups, suggesting that completing more exercises increased the probability of belonging to the High-responders group. In contrast, completing fewer exercises was associated with a greater probability of belonging to the Partial-responders group (with negligible effects on the No-responders group).

Table 2 is a full regression table resulting from multinomial logistic regression with children’s characteristics (i.e., age, reading difficulty, initial reading accuracy, initial reading speed, number of exercises) as predictors of the membership to the No, Partial, and High responder clusters. Regression coefficients (β) are the core parameters estimated by a logistic regression model. They quantify how much and in what direction a predictor variable influences the outcome.

As shown in Table 2, the statistically significant predictors are as follows:

- Age influences membership in the No-Responder versus High-Responder cluster (β = −0.39) and in the Partial-Responder versus High-Responder cluster (β = −0.48). The negative s ign indica te s an inverse rela tionship, meaning that younger age is predictive of belonging to the High-Responder cluster.
- Reading difficulty influences membership in the No-Responder versus High-Responder cluster (β = 0.52) and in the Partial-Responder versus High-Responder cluster (β = 0.61).
- Initial accuracy influences membership in the Partial-Responder versus High-Responder cluster (β = 0.26).
- Number of exercises influences membership in the Partial-Responder versus High-Responder cluster (β = 0.36).

In terms of cross-validation, the confusion matrix on the training set (n = 525) confirms that the model predominantly predicts the High-Responder cluster (362 true positives), while struggling with No-Responder and Partial-Responder clusters (0 and 2 true positives, respectively).

### Parameter Clusters Differences

A mixed-design ANOVA was conducted on reading speed with B–E training as the within-subjects factor and reading unit (RU-cluster) cluster as the between-subjects factor. The analysis revealed a significant interaction, but a negligible effect size (F _(3,6579)_ = 14.80, p < .001, partial η^2^ = .000). An analysis of the post hoc results shows that the most pronounced improvement (mean beginning-end training difference in reading speed = −0.367 syll/sec, SE = 0.014, df = 6,579, t = −26.705, p <.001) occurs in the cluster of children who begin treatment with syllables and, during the central period, transition to words. For the other three clusters, the improvement ranges from 0.254 to 0.282 (SE = 0.034, df = 6,579, t = −7.482, p <.001) to −0.282 syll/sec (SE = 0.005, df = 6,579, t = −54.645, p <.001).

**Figure 9:**
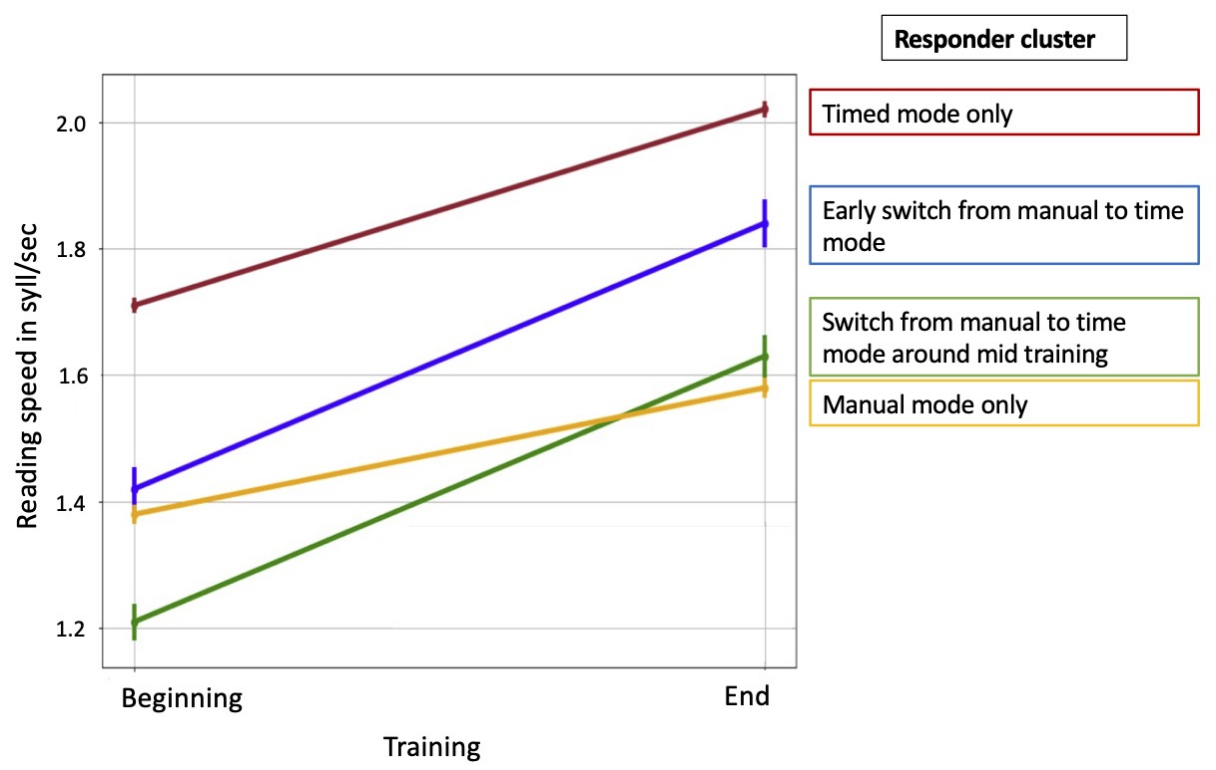
Means and Standard Errors of the Reading Speed Scores Obtained at the Beginning and End of the Training in the Four PM Clusters

A mixed-design ANOVA was conducted on reading speed, with B–E as the within-subjects factor and progression mode cluster (PM-cluster) as the between-subjects factor. The analysis revealed a significant interaction with a medium effect size (F _(3,6579)_ = 144.00, p < .001, partial η^2^ = .061). Post-hoc comparisons (Tukey test) showed that children who switched from manual to timed mode during training (clusters 1 and 2) demonstrated a substantially greater improvement in reading speed than those who remained in manual mode (cluster 3) throughout, and a slightly greater improvement than those who used the timed mode from start to end (see Table 2 for post-hoc tests and Figure 9 for plot means).[Table t3-ijt-18-1-6735]

## Discussion

The study had three main objectives. The first was to identify different response trajectories based on the learning curve and the performance gains from the beginning to the end of the intervention. The second was to examine the role of individual characteristics, particularly age, initial reading difficulty level, and number of completed exercises, in differentiating response clusters. Finally, the third objective was to assess the impact of the training parameters, specifically reading units (morphemes, syllables, and words) and exercise progression type (automated vs manual), on the efficacy of the intervention.

Cluster analyses on reading speed (syllables per second) across a two-month training period identified eight distinct learning curves. Visual inspection showed that in four clusters (LC-clusters 1–4), referring to a small proportion of subjects each (for a total of 29.7%), learning curves did not show an evident increase over time; one cluster (LC-cluster 5: 10.22%) exhibited a curve with an initial improvement followed by a plateau; and three clusters (LC-Clusters 6–8), covering the largest proportion of subjects (for a total of 60.1%), displayed a progressive increase over time.

This pattern of findings broadly confirms previous studies on the effectiveness of multicomponent and adaptive teleintervention, such as Reading Trainer (e.g., Pecini et al., 2018; Tucci et al., 2015). Additionally, it highlights for the first time the existence of high variability in the response curve to the intervention despite its global efficacy. This type of analysis was possible thanks to the use of telemedicine-based interventions (allowing the continuous recording of the response on large samples of subjects) together with the potential of machine learning that automatically identifies homogeneous patterns of data that would not be detectable manually. A more refined distinction in intervention responses, beyond pre- vs. post-assessment differences, is relevant in developmental interventions, since children’s responses are typically more variable, less consistent, and less systematic than those of adults (Capodieci et al., 2023; Dvorsky et al., 2021). Additionally, a more nuanced understanding of individual progress throughout the intervention enables timely adjustments and individualized support, an approach challenging to implement with traditional paper-and-pencil approaches (Hadders-Algra, 2021).

To verify and quantify the improvements associated with the different learning curves explained by the 8 LC-clusters, inferential analyses were applied to compare the clusters on the mean reading speed and accuracy at the beginning and end of the intervention. The evaluation of reading speed showed the existence of two clusters (LC-clusters 1–2) characterized by non-significant changes (*No-responders*), two clusters (LC-clusters 3–4) with significant differences between the beginning and the end of the intervention but small changes in terms of effect size (*Partial-responders*), and four clusters (LC-clusters 5–8) characterized by significant and marked improvements (*High-responders*). Thus, integrating the learning curve with the differences in the improvement from the beginning to the end of the training allows a better identification of different groups of responders to the reading intervention. These differences can be crucial to consider, contingent upon the therapy’s parameters and the individual’s factors.

Regarding reading accuracy, inferential statistics showed the absence of significant differences across the 8 LC-clusters. This outcome was partly expected, given that progression in training was contingent upon reaching an accuracy threshold before a given speed was targeted. In fact, accuracy rates in our sample were consistently high (i.e., over 95%), making cluster differences less likely. Future research employing interventions that simultaneously target speed and accuracy may shed light on the dynamic interplay between decoding fluency and accuracy.

Regarding the second objective, predictive analyses confirmed the role of children’s characteristics and behavior during training on the different groups of responders. Age, baseline difficulty level, and number of completed exercises were all significant predictors. Specifically, younger children, those with greater initial difficulties and those completing a higher number of exercises were more likely to belong to the *High-responder* clusters. This finding allows us to generalize to teleintervention training the previous evidence on the efficacy of reading fluency interventions, which indicates that more intensive, early, and individualized interventions are associated with greater benefits, particularly for children with more pronounced reading difficulties (Fraga-Gonzales et al., 2020; Galuschka et al., 2014; Hall et al., 2023; Lovett et al., 2017). However, it should be noted that the regression model predominantly predicts the *High Responders* group; therefore, the study results do not help us identify the characteristics of those who struggle to respond to the intervention. Furthermore, certain variables are correlated with one another, making it challenging to determine their directionality (e.g., number of exercises and session duration). Thus, to attribute a causal role to any of the identified predictors, the findings of this retrospective study should be integrated with those from controlled experimental designs.

Finally, regarding the third objective of the study, the results emphasized the impact of training characteristics on training efficacy. The type of reading unit used during the exercises significantly affected the differences observed between the beginning and final phases of the intervention. Children who were initially trained on syllable units and later on word units demonstrated greater improvement than those who consistently trained on syllables, morphemes, or words throughout the entire training period. This finding can be understood within the general framework of the dual-route model of reading (Coltheart et al., 2001). Developmental longitudinal studies stemming from this theoretical perspective indicate that, in regular orthographies such as Italian, phonological reading develops from the aloud conversion of small orthographic units to aloud conversion of whole strings, a process underlying the systematic expansion of lexical reading (Orsolini et al., 2006).

Consistently, cross-linguistic studies highlight that this type of reading acquisition is characteristic of languages with regular orthographies, such as German (Wimmer & Goswami, 1994), Welsh (Ellis & Hooper, 2001), or Italian (Marinelli et al., 2016), when compared to a very opaque orthography, such as English. Thus, in Italian, reading in the early stages of acquisition (e.g., early elementary school) is primarily influenced by non-lexical factors, such as word length (Zoccolotti et al., 2005). Only later (around third grade) do lexical influences, such as the frequency effect, become clearly evident (Zoccolotti et al., 2009). Seen in this developmental perspective, it seems reasonable that training that first develops the phonological route (syllables) and then the lexical route (words) is more effective than focusing on just one route at a time, particularly in regular orthographies. However, it should be noted that the effect size of the type of reading unit was very small. Thus, the statistical significance could be attributed to the large sample of data analyzed in the present study. Furthermore, it cannot be excluded that the choice to switch from one reading unit to another was a consequence rather than a cause of the improvements obtained by the child. At any rate, the indication that a progression from a training focused initially on syllable units and subsequently moving to word units marks a particularly effective acquisition stands as an interesting finding which appears worth pursuing in further targeted research.

Furthermore, the progression mode yielded a significant effect on training efficacy, with a medium size effect on the differences between the beginning and the end of the training. The most marked improvements occurred when exercises transitioned from manual to automated timing modes, compared to when the child was trained separately on the manual or automated mode for the entire training. As paced reading can foster, more than manually controlled reading, automatic access to the lexical route (Lorusso et al., 2005; Luci et al., 2022), this result may also reflect that the switch in this treatment parameter represents an optimal balance to enable children to enhance reading fluency.

Although further research is needed to clarify how these training parameters interact with other individual and treatment-related factors, the present findings call the clinician’s attention to the importance of exercise adaptability in teleinterventions for decoding skills, as a means of tailoring training to children’s performance and optimizing the interplay between accuracy and speed.

### Limits and Future Directions of Research

The nature of the analyzed dataset also highlights some limitations of the present study.

Critically, regarding sample selection, this study prioritizes ecological validity by evaluating training responses within routine clinical practice rather than under strictly controlled experimental conditions. A significant advantage of this approach is that, by not applying rigid inclusion or exclusion criteria, we captured a realistic representation of the clinical population. This high level of variability provided an ideal foundation for the machine learning approach, which is specifically designed to disentangle complex, non-linear patterns within ‘real-world’ datasets that traditional group-based analyses might overlook. However, this methodological choice also introduces certain limitations. The resulting sample is highly heterogeneous, encompassing a broad range of profiles, particularly in terms of specific diagnoses and potential comorbidities. Although we controlled for key variables such as age and baseline reading difficulty, this diversity must be carefully considered when interpreting the findings, as it may limit the direct comparability of our results with studies conducted on highly selected clinical samples.

Furthermore, we did not have access to children’s pre- and post-reading evaluations based on standardized clinical instruments. Thus, we derived the measures of reading speed and accuracy from the output of the training application itself, i.e., the reading of the texts used during the intervention. To partially address this issue, performance was analyzed using difference scores rather than absolute values and transformed into z-scores relative to age-expected reading speeds. This procedure enabled meaningful comparisons supporting global analyses of the dataset. However, this characteristic also limits the comparability of the present results with those reported in the broader literature as well as the generalizability of the results to everyday contexts.

While the availability of a large dataset has helped highlight areas that warrant further investigation, the use of smaller but clinically better-defined samples of children may be instrumental in addressing these questions. As stated above, there are considerable individual differences in the learning trajectories as a function of the reading training. It would be particularly relevant to acquire information on the individual cognitive characteristics which are associated with such differential outcomes.

The low degree of control over the intervention’s characteristics may also represent a limitation of the study, restricting the interpretability of the findings. Specifically, session duration, the type of technology utilized, and the integration of remote versus in-person modalities represent variables that could influence the intervention’s effectiveness and should be controlled for in future studies.

A further consideration concerns the scope of our analysis. The present study does not aim to validate the clinical efficacy of the intervention *per se*, but rather to demonstrate how machine learning algorithms can identify distinct developmental trajectories and ‘improvement profiles’ within a large dataset. From this perspective, the choice to focus on a single, well-defined pillar of reading, decoding, was intentional. By isolating decoding from broader processes like text comprehension (Gough & Tunmer, 1986; Scarborough, 2001), we were able to reduce the ‘noise’ in the data, allowing the machine learning models to detect how individual variables influence the automatization of word recognition more accurately. However, we acknowledge that this focus represents a boundary. While the machine learning approach effectively identifies who benefits most from decoding training, it does not account for the complex interplay between decoding and language comprehension that characterizes the full reading system. Future research employing machine learning on multi-dimensional datasets, including text comprehension and its underpinnings, would be instrumental in providing a more holistic view of reading profiles.

Finally, a further methodological limitation concerns the K-means algorithm, which assumes spherical cluster shapes and equal variance across clusters. Future studies should compare these results with hierarchical clustering or Gaussian mixture models to further verify the robustness of the identified trajectories.

## Conclusions

This study successfully leveraged unsupervised machine learning on a large-scale, continuous telerehabilitation dataset to identify distinct learning trajectories in children with decoding fluency difficulties. It revealed significant variability in responses that traditional pre-post designs had previously failed to detect. The automatic identification of homogeneous patterns within the data provided valuable insights into predictive individual factors, as well as tailored intervention parameters linked to positive learning curves and substantial improvements in reading speed. Although further investigation is needed into other pre-existing cognitive factors, the findings emphasize the value of applying artificial intelligence to develop algorithms that learn from data. This approach can enhance adaptive teleinterventions for optimizing reading strategies and offer clinicians actionable evidence for personalizing training protocols.

## Figures and Tables

**Table 1:** Differences in Reading Speed (syll./sec.) between the Beginning and the End of the Training and the Results of the Paired Post-hoc Tests

LC-Cluster	Mean beginning-end difference (reading speed)	SE	t	df	p*	Effect size (d)
1	0.009	0.013	0.69	6575	1.00	0.008
2	0.019	0.011	1.66	6575	0.96	0.021
3	−0.125	0.011	−11.31	6575	<.001	−0.140
4	−0.130	0.011	−12.04	6575	<.001	−0.146
5	−0.247	0.010	−24.50	6575	<.001	−0.304
6	−0.264	0.008	−32.03	6575	<.001	−0.407
7	−0.441	0.006	−69.50	6575	<.001	−0.907
8	−0.417	0.006	−67.22	6575	<.001	−0.858

*Note*.

* Tukey test

**Table 2:** Children’s Characteristics as Predictors of Membership to the No, Partial, and High Responder Clusters: β Coefficients from Multinomial Logistic Regression

Comparison	Predictor	Coefficient (β)	Standard Error	z-value	p-value
No responder vs. High responder	age	−0.39	0.18	−2.15	0.03
Partial responder vs. High responder	age	−0.48	0.15	−3.17	0.00
No responder vs. High responder	reading difficulty	0.52	0.18	2.86	0.00
Partial responder vs. High responder	reading difficulty	0.61	0.15	4.05	0.00
No responder vs. High responder	initial accuracy	0.13	0.07	1.83	0.07
Partial responder vs. High responder	initial accuracy	0.26	0.06	4.25	0.00
No responder vs. High responder	initial speed	0.20	0.22	0.93	0.35
Partial responder vs. High responder	initial speed	0.15	0.18	0.87	0.39
No responder vs. High responder	number_of_exercises	0.00	0.09	0.01	0.99
Partial responder vs. High responder	number_of_exercises	0.36	0.07	5.04	0.00
No responder vs. High responder	Intercept	0.32	0.08	3.81	0.00
Partial responder vs. High responder	Intercept	1.71	0.07	24.83	0.00

**Table 3 t3-ijt-18-1-6735:** Post-hoc Tests of the Mixed-design ANOVA B–E vs PM-cluster

PM-Cluster	Mean Beginning-End training difference (reading speed)	SE	t	df	p*	Effect size (d)
Manual-timed 25–50 days	−0.426	0.015	−28.35	6579	<.001	−0.351
Manual-timed 6–23 day	−0.422	0.015	−28.24	6579	<.001	−0.347
Manual	−0.192	0.006	−31,61	6579	<.001	−0.394
Timed	−0.309	0.004	−64.75	6579	<.001	−0.951

*Note*.

* Tukey test
